# Effect of repetitive lysine–tryptophan motifs on the bactericidal activity of antimicrobial peptides

**DOI:** 10.1007/s00726-012-1388-6

**Published:** 2012-08-23

**Authors:** Ramamourthy Gopal, Chang Ho Seo, Peter I. Song, Yoonkyung Park

**Affiliations:** 1 Research Center for Proteineous Materials (RCPM), Chosun University, Kwangju, South Korea; 2 Department of Bioinformatics, Kongju National University, Kongju, South Korea; 3 Department of Dermatology, University of Arkansas for Medical Sciences, Little Rock, AR USA; 4 Department of Biotechnology, Chosun University, Kwangju, South Korea

**Keywords:** Lysine, Tryptophan, Amidation, Antimicrobial peptides, Membrane permeabilization, Bacterial agglutination

## Abstract

Previous studies identified lysine- and tryptophan-rich sequences within various cationic antimicrobial peptides. In the present study, we synthesized a series of peptides composed of lysine (K)-tryptophan (W) repeats (KW)_*n*_ (where *n* equals 2, 3, 4 or 5) with amidation of the C-terminal to increase cationicity. We found that increases in chain length up to (KW)_4_ enhanced the peptides’ antibacterial activity; (KW)_5_ exhibited somewhat less bactericidal activity than (KW)_4_. Cytotoxicity, measured as lysis of human red blood cells, also increased with increasing chain length. With (KW)_5_, reduced antibacterial activity and increased cytotoxicity correlated with greater hydrophobicity and self-aggregation in the aqueous environment. The peptides acted by inducing rapid collapse of the bacterial transmembrane potential and induction of membrane permeability. The mode of interaction of the peptides and the phosphate groups of lipopolysaccharide was dependent upon the peptides’ ability to permeate the membrane. Longer peptides [(KW)_4_ and (KW)_5_] but not shorter peptides [(KW)_2_ and (KW)_3_] strongly bound and partially inserted into negatively charged, anionic lipid bilayers. These longer peptides also induced membrane permeabilization and aggregation of lipid vesicles. The peptides had a disordered structure in aqueous solution, and only (KW)_4_ and (KW)_5_ displayed a folded conformation on lipid membranes. Moreover, (KW)_4_ destroyed and agglutinated bacterial cells, demonstrating its potential as an antimicrobial agent. Collectively, the results show (KW)_4_ to be the most efficacious peptide in the (KW)_*n*_ series, exhibiting strong antibacterial activity with little cytotoxicity.

**Electronic supplementary material:**

The online version of this article (doi:10.1007/s00726-012-1388-6) contains supplementary material, which is available to authorized users.

## Introduction

Antimicrobial peptides (AMPs) are small proteins (~40 amino acids residues) (Brown and Hancock 2006) that can be used as potent weapons against infectious microbes, including multidrug-resistant bacteria (Hancock 2001). Indeed, AMPs constitute key parts of the innate immune systems of numerous organisms, including amphibians, and mammals. Because conventional antibiotics have specific intracellular targets, bacteria are able to develop compensatory mechanisms that render them resistant (Alanis 2005; Tenover 2006). In contrast, AMPs bind to the bacterial cell membrane and perturb its structure. This modus operandi makes AMPs impervious to bacterial resistance, since developing resistance would require microbes to alter their entire membrane lipid composition. Consequently, AMPs are attractive therapeutic candidates for protection against multidrug-resistant microbes (Fjell et al. 2011; Brogden and Brogden 2011; Giuliani and Rinaldi 2011).

AMPs adopt both *α*-helical and β-sheet secondary structures within hydrophobic environments (Powers and Hancock 2003). Despite differences in their secondary structures, most antimicrobial peptides share certain features, including cationicity, hydrophobicity and amphipathicity, which are essential for disruption of bacterial membranes. The cationicity of AMPs is attributable to the presence of numerous Arg and Lys residues, while amphipathicity reflects the presence of polar amino acids and non-polar, hydrophobic residues on opposite sides of the peptide secondary structure (Epand et al. 2003; Gopal et al. 2009; Kiyota et al. 1996; Song et al. 2005). The initial interaction between the bacterial membrane and an AMP is electrostatic in nature, since the bacterial membrane consists of anionic lipids such as phosphatidylglycerol and cardiolipin as well as the zwitterionic lipid phosphatidylethanolamine (Zilberstein et al. 1979; Lugtenberg and Van Alphen 1983). Once the polar side of the AMP, which is enriched with charged residues, binds to the anionic bacterial membrane, the hydrophobic region of the peptide attaches to the hydrophobic core of the lipid bilayer, thereby causing a partition.

Although charge and amphipathicity greatly influence the activity of AMPs, naturally occuring AMPs also differ greatly in length, making it very difficult to correlate chain length and antimicrobial activity. Because knowing the optimal length of AMPs could facilitate de novo design of AMPs (Deslouches et al. 2005), we designed and synthesized a series of peptides of different length, and then determined their modes of action using lipid bilayer and bacterial membrane models. We found that the length of the peptide chain greatly influences target selection, permeabilization of the bacterial membrane, and agglutination of bacterial cells.

## Materials and methods

### Materials

Rink amide 4-methylbenzhydrylamine resin, fluoren-9-ylmethoxycarbonyl (Fmoc) amino acids and other reagents for peptide synthesis were purchased from Calbiochem-Novabiochem (La Jolla, CA, USA). Acrylamide was from Sigma-Aldrich (St. Louis, MO, USA). Cholesterol (CH, from porcine liver), l-*α*-phosphatidylethanolamine (PE, from *E. coli*), egg yolk l-*α*-phosphatidylcholine (PC) and l-*α*-phosphatidylglycerol (PG, from *E. coli*) were from Avanti Polar Lipids Co. (Alabaster, AL, USA). Calcein, 5-carboxytetramethylrhodamine (TAMRA), 3,3′-diethylthiodicarbocyanine iodide (DiSC_3_-5) and SYTOX Green were from Molecular Probes (Eugene, OR, USA). All other reagents were of analytical grade. Buffers were prepared using double distilled water (Millipore Co.).

### Bacterial strains

*Escherichia coli* (KCTC 1682), *Salmonella typhimurium* (KCTC 1926), *Pseudomonas aeruginosa* (KCTC 1637), *Staphylococcus aureus* (KCTC 1621), *Bacillus subtilis* (KCTC 1918), and *Listeria monocytogenes* (KCTC 3710) were obtained from the Korean Collection for Type Cultures (KCTC). Drug-resistant *E. coli* strains (CCARM 1229 and CCARM 1238), *S. typhimurium* strains (CCARM 8007, CCARM 8009, and CCARM 8013), and *S. aureus* strains (CCARM 3089, CCARM 3090, CCARM 3108, CCARM 3114, and CCARM 3126) were from the Culture Collection of Antibiotic-Resistant Microbes (CCARM) at Seoul Women’s University, Korea.

### Peptide synthesis and purification

The peptides KWKW-NH_2_ [(KW)_2_], KWKWKW-NH_2_ [(KW)_3_], KWKWKWKW-NH_2_ [(KW)_4_], and KWKWKWKWKW-NH_2_ [(KW)_5_] were synthesized using the solid-phase method with Fmoc chemistry on a solid support of rink amide 4-methylbenzhydrydrylamine resin. Thereafter, 0.1 M *N*-hydroxy benzotriazole (HOBt), 0.45 M 2-(1H-benzotriazole-1-yil)-1,1,3,3-tetramethyluroniumhexafluorophosphate (HBTU) in dimethylformamide (DMF) and 2 M *N*,*N*-diisopropyl ethylamine (DIEA) in *N*-methylpyrrolidone (NMP) were used as coupling reagents, and a tenfold excess of Fmoc-amino acid was added during every coupling cycle. After a final deprotection step using a solution of 20 % piperidine in DMF, cleavage was carried out using a mixture of TFA/water/triisopropylsilane (90:5:5) for 2 h at room temperature. The crude peptides were repeatedly extracted with diethyl ether and then purified using reverse phase preparative HPLC on a Vydac C_18_ column (4.6 × 250 mm, 300 Å, 5 μm). The molecular masses of the peptides were confirmed using a matrix-assisted laser desorption ionization mass spectrometer (MALDI II, Kratos Analytical Ins.).

### Antibacterial activity

The antibacterial activities of the peptides against Gram-negative, Gram-positive and antibiotic-resistant bacterial strains were evaluated using the microbroth dilution method (Park et al. 2003). Aliquots of culture medium containing bacterial suspensions in mid-logarithmic phase at a concentration of 2 × 10^5^ colony forming units (CFU)/ml were added to each well of a multiwell plate containing peptide solution serially diluted 2× in 10 mM sodium phosphate buffer (pH 7.2) or phosphate-buffered saline (PBS, 1.5 mM KH_2_PO_4_, 2.7 mM KCl, 8.1 mM Na_2_HPO_4_, 150 mM NaCl, pH 7.2). Growth inhibition was evaluated by measuring the absorbance at 620 nm using a Versa-Max microplate Elisa Reader (Molecular Devices Co., Sunnyvale, CA, USA) after 24 h of incubation at 37° C. The minimum inhibitory concentration (MIC) was defined as the minimal peptide concentration that completely inhibited bacterial growth. All MIC measurements were the average of 3–4 independent experiments.

### Human red blood cell (hRBC) hemolysis

Hemolytic activities of the peptides were assessed using heparinized hRBCs collected from healthy donors. Fresh hRBCs were washed three times by centrifugation at 800×*g* for 10 min in PBS (pH 7.2) and then resuspended in PBS. The peptides were then dissolved in PBS and added to 100 μl of stock hRBCs suspended in PBS (final RBC concentration, 4 % v/v), after which the samples were incubated with gentle agitation for 60 min at 37° C before centrifugation for 10 min at 800×*g*. Absorbance of the supernatants was recorded at 414 nm. In addition, control samples for zero hemolysis and 100 % hemolysis were incubated with PBS (*A*_blank_) or 0.1 % Triton X-100 (*A*_triton_), respectively. Melittin was used as a reference hemolytic peptide. Percent hemolysis was calculated according to the following equation:

$$ \%\, {\text{Hemolysis}} = [(A_{\text{sample}} -A_{\text{blank}})/(A_{\text{triton}} - A_{\text{blank}})] \times 100.  $$

Each measurement was conducted in triplicate (Kim et al. 2011).

### Computational modeling

The structures of the peptide models were built using ChemOffice Desktop 2004 for Windows (CambridgeSoft (CS) Corporation, MA, USA). The structures of linear peptides were generated and the energies minimized using Chem3D and the molecular mechanics method (MM2) (Burkert and Allinger 1982). The sequences of the peptides were initially added using standard ChemDraw Ultra, after which the N-and C-termini of the peptide groups were charged by changing the balances. Amidation of the C-terminus of each peptide was carried out using Chem3D. Energy minimizations were run for all of the peptides using the Chem3D and MM2 methods. Finally, optimized peptides were imported into the Pymol program for visualization and figure generation (DeLano 2002).

### Aggregation states of peptides in aqueous solution

We compared the aggregation states of peptides in water and PBS by examining Trp fluorescence as a function of peptide concentration from 1 to 18 μM. Changes in emission were recorded using a spectrofluorometer (Perkin-Elmer LS55, Mid Glamorgan, UK) at an excitation wavelength of 280 nm and an emission wavelength of 300–400 nm.

CD spectroscopy was used to determine the aggregation states of the peptides in PBS at various concentrations ranging from 25 to 150 μM. The CD spectra were recorded between 190 and 250 nm on a Jasco 810 spectropolarimeter (Jasco, Tokyo, Japan) equipped with a temperature control unit. The recordings were made using a quartz cell with a 0.1-cm path length at 25° C. At least four scans over the 190–250 nm wavelength range were conducted.

### Confocal laser-scanning microscopy (CLSM)

Fluorescently labeled (KW)_4_ peptide was used for the CLSM study. Fluorescent labeling of the N-termini of the peptides was accomplished using TAMRA in DMF (3–4 eq.) containing 5 % (v/v) DIEA (Ghosh et al. 1997).

*Escherichia coli* CCARM 1229 or *S. aureus* CCARM 3090 cells in mid-logarithmic phase were harvested by centrifugation, washed and re-suspended in PBS (pH 7.2) to a concentration of 2 × 10^5^ CFU/ml. TAMRA-labeled peptide was then added to 100 μl of the cell suspension to a concentration of 12.5 μM, after which the cells were incubated at 37° C for 10 min, pelleted by centrifugation at 3,000×*g* for 5 min, and washed three times with PBS. The action site of TAMRA-labeled (KW)_4_ was then examined using an inverted LSM510 laser-scanning microscope (Carl Zeiss, Göttingen, Germany).

### Dansyl polymyxin B (PMB) displacement assay

The binding affinity of each peptide for lipopolysaccharide (LPS) was determined using dansyl PMB displacement assays (Moore et al. 1986). Dansyl PMB (4 μM/ml) and *P. aeruginosa* LPS (9 μg/ml) were mixed in 1 ml of 5 mM HEPES (pH 7.2) for optimal fluorescence. Reductions in fluorescence were used to calculate the percent displacement of dansyl PMB after peptide treatment. Buffer blank was subtracted from the peptide spectra. Spectra were recorded on a Perkin-Elmer LS-50B spectrofluorimeter using a cuvette with of 1-cm path length. The excitation and emission wavelengths were 340 and 485 nm, respectively.

### Membrane depolarization

Peptide-induced membrane depolarization was examined using Gram-positive and Gram-negative bacteria as described previously (Papo et al. 2002; Sal-Man et al. 2002). Briefly, *E. coli* CCARM 1229 and *S. aureus* CCARM 3090 were grown to mid-logarithmic phase at 37° C with gentle agitation. The cells were then washed twice in buffer (20 mM glucose, 5 mM HEPES, PH 7.3) and re-suspended to an OD_600_ of 0.05 in similar buffer containing 0.1 M KCl. The bacterial cells were then incubated with 1 μM DiSC_3_-5 until the fluorescence reached a stable baseline. After addition of the indicated concentrations of peptide, changes in fluorescence were measured using an excitation wavelength of 622 nm and emission wavelength of 670 nm. The fluorescence of 0.1 % Triton X-100-treated cells served as a positive control for maximum depolarization.

### SYTOX Green uptake

Cultured cells (*E. coli* CCARM 1229 and *S. aureus* CCARM 3090) were re-suspended (2 × 10^7^ CFU/ml) in PBS and incubated with 1 μM SYTOX Green for 10 min in the dark. Peptides (12.5 μM) were then added to the cell suspension, and the fluorescence was monitored for 60 min at an excitation wavelength of 485 nm and emission wavelength of 520 nm.

### Kinetic studies

The kinetics of the bactericidal activity of peptides were evaluated using *E. coli* CCARM 1229 and *S. aureus* CCARM 3090. Mid-logarithmic growth phase bacteria (2 × 10^5^ CFU/ml) were incubated with peptides at 1× or 2× the MIC at 37° C. Aliquots were removed at the indicated times, appropriately diluted, and plated on LB agar plates. CFUs were counted after 16 h of incubation at 37° C. All measurements were carried out in triplicate.

### Preparation of small and large unilamellar vesicles (SUVs and LUVs)

SUVs were prepared by sonication of the required amount of PE:PG (7:3, w/w) or PC:CH (10:1, w/w). Dry lipids were dissolved in chloroform in a glass vessel, after which the solvent was removed using a stream of nitrogen gas. Any remaining trace amounts of organic solvent were then completely removed by lyophilization overnight, and the remaining dry lipid film was resuspended in PBS at (pH 7.2) with gentle vortex mixing. The lipid suspensions were then sonicated at 40° C until clear using a bath-type ultrasonicator and extruded 14 times using an Avanti Mini-Extruder (Avanti Polar Lipids Inc., Alabaster, AL, USA) with 0.05 μm polycarbonate filters.

LUVs were prepared using the freeze–thaw method (Mayer et al. 1986). Briefly, dry lipid films were resuspended in 1–2 ml of appropriate buffer by vortexing. LUVs were prepared through nine freeze–thaw cycles in liquid nitrogen, followed by incubation in a water bath at 50° C. The suspensions were then extruded 14 times through 0.2-μm polycarbonate membranes, and lipid concentrations were determined using standard phosphate assays (Stewart 1980).

### Calcein leakage from liposomes

PE:PG (7:3, w/w) and PC:CH (10:1, w/w) LUVs with entrapped calcein were prepared by vortexing the dried lipids in a dye buffer solution (70 mM calcein, PBS, pH 7.4). The resultant suspension was then subjected to nine cycles of freeze–thaw in liquid nitrogen, followed by separation of the calcein-containing vesicles from free calcein using gel filtration chromatography on a Sephadex G-50 column. LUVs with entrapped calcein in a suspension containing 2.5 μM lipid were then incubated for 25 min with various concentrations of peptide (0.03–0.25 μ). The release of fluorescent calcein was assessed using a spectrofluorometer at an excitation wavelength of 480 nm and an emission wavelength of 520 nm. Complete (100 %) release was induced by addition of 0.1 % Triton X-100; spontaneous leakage was negligible. All experiments were conducted at 25° C, and the apparent percentage of released calcein was calculated using the following equation (Matsuzaki et al. 1999):

$$ {\text{Release}} \, (\%) = 100 \times (F - F_{o})/(F_{t} - F_{o})$$

where *F* and *F*_*t*_ are the fluorescence intensity before and after the addition of detergent, respectively, and *F*_*o*_ is the fluorescence of intact vesicles.

### Liposome aggregation

The aggregation of lipid vesicles was monitored through visible absorbance measurements. Peptides (5, 10, 20, and 40 μM) in PBS (pH 7.2) were added to a suspension of 400 μM PE:PG LUVs (7:3, w/w). Absorbance was measured at 405 nm using a microplate Autoreader before and after the addition of the peptides (Oren et al. 1999). Increased absorbance indicated liposomal aggregation.

### Trp fluorescence and acrylamide quenching assays

The fluorescence emission spectrum of Trp within the peptides was monitored using a spectrofluorometer in the presence of PE:PG (7:3, w/w) or PC:CH (10:1, w/w) SUVs, which were used to minimize differential light scattering effects (Mao and Wallace 1984). Each peptide was added to 1 ml of 200 μM liposomes, after which the peptides and liposomes (molar ratio 1:100) were allowed to interact for 10 min at 25° C. The fluorescence was then measured at an excitation wavelength of 280 nm and an emission wavelength from 300 to 400 nm.

Fluorescence quenching experiments were conducted using acrylamide as the quencher. The concentration of acrylamide in the cuvette ranged from 0.04 to 0.20 M. The effect of acrylamide on the fluorescence of each peptide was analyzed using the Stern–Volmer equation

$$ F_{o}/F= 1+K_{\text{sv}} (Q)$$

where *F*_0_ and *F* are the fluorescence intensities in the presence or absence of acrylamide, respectively, *K*_SV_ is the Stern–Volmer quenching constant and (*Q*) is the concentration of acrylamide.

### CD spectroscopy

The CD spectra for 50 μM peptide dissolved in PBS alone (pH 7.2), PBS containing 1 mM PE:PG (7:3, w/w) vesicles, or PBS containing 1 mM PC:CH (10:1, w/w) vesicles were scanned in the presence or absence of LPS (0.1 %) dissolved in PBS. The CD data represent the average values of three separate recordings with four scans per sample.

### Scanning electron microscopy (SEM)

Mid-logarithmic phase cultured *E. coli* CCARM 1229 cells were re-suspended at 2 × 10^7^ CFU/ml in PBS (pH 7.2) and incubated with 12.5 μM (KW)_4_ for 1 h at 37° C. The bacteria were then pelleted by centrifugation at 3000×*g* for 5 min and washed twice in PBS. After removing the supernatants, the pellets were fixed for 3 h at 4° C in 500 μl of 5 % (v/v) glutaraldehyde in 0.2 M sodium-cacodylate buffer (pH 7.4). The samples were then extensively washed with 0.1 M sodium-cacodylate buffer and treated with 1 % (w/v) osmium tetroxide in 0.1 M sodium-cacodylate buffer for 1 h at 4° C in the dark. Thereafter, the bacteria were washed twice with 5 % (w/v) sucrose in the same buffer, and dehydrated in a sequential 20, 40, 60, 80, 95 and 100 % ethanol series. After lyophilization and coating, the samples were examined in a scanning electron microscope (Hitachi S-2400N, Japan).

### Bacterial agglutination assay

Overnight cultures of *E. coli* CCARM 1229, *S. aureus* CCARM 3090 and *P. aeruginosa* were washed with 10 mM sodium phosphate (pH 7.4) containing 10 % TSB media. After adjusting the concentration to 2 × 10^8^ CFU/ml, 100 μl aliquots of the bacterial suspension were incubated with 100 μl of peptide solution (6.25–50 μM) in a polystyrene plate for 1 h at 37° C. The bacteria were then stained for 10 min with 1 % (w/v) crystal violet. After the supernatants containing non-aggregated cells were carefully removed, each well was photographed using an Olympus Inverted Microscope (Olympus 1X71, Tokyo, Japan) equipped with digital camera (Olympus DP71).

## Results

### Effects of hydrophobicity and length on the antibacterial and hemolytic activities of (K–W)_*n*_ peptides and computer modeling of peptide structures

The observed molecular weights of the synthetic (KW)_*n*_ peptides were well matched to the calculated values. To determine the antibacterial and hemolytic activities of the peptides, we used RP-HPLC to assess the level of hydrophobicity of the synthetic peptides (Table 1). We found that the retention times, and thus the relative hydrophobicity, of the (KW)_*n*_ peptides were in the order (KW)_2_ < (KW)_3_ < (KW)_4_ < (KW)_5_, indicating that the hydrophobicity of the synthetic peptides increased with the addition of KW motifs.

**Table 1:** MICs of peptides used against bacterial strains

Microorganisms	(KW)_2_	(KW)_3_	(KW)_4_	(KW)_5_	Melittin	Ampicillin	Oxacillin
*MIC (μM)*							
Gram (−) bacteria							
*E. coli*	>200 (>200)	50 (200)	6.25 (12.5)	50 (12.5)	1.56 (1.56)	50	–
*S. typhimurium*	50 (200)	3.12 (6.25)	1.56 (1.56)	6.25 (3.12)	0.39 (0.39)	25	–
*P. aeruginosa*	>200 (>200)	12.5 (25)	3.12 (3.12)	25 (6.25)	3.12 (3.12)	–	–
Gram (+) bacteria							
*S. aureus*	>200 (>200)	50 (200)	6.25 (12.5)	25 (12.5)	1.56 (1.56)	–	12.5
*B. subtilis*	>200 (>200)	100 (200)	6.25 (12.5)	12.5 (12.5)	1.56 (3.12)	–	–
*L. monocytogenes*	200 (>200)	12.5 (25)	3.12 (3.12)	3.12 (3.12)	1.56 (1.56)	–	–
Resistant strains							
*E. coli* CCARM 1229^a^	>200 (>200)	25 (100)	6.25 (12.5)	50 (12.5)	3.12 (3.12)	>200 (>200)	–
*E. coli* CCARM 1238^a^	>200 (>200)	50 (200)	12.5 (12.5)	50 (12.5)	1.56 (1.56)	>200 (>200)	–
*S. typhimurium* CCARM 8007^b^	>200 (>200)	50 (200)	12.5 (12.5)	25 (12.5)	6.25 (12.5)	>200 (>200)	–
*S. typhimurium* CCARM 8009^b^	>200 (>200)	50 (200)	6.25 (12.5)	25 (12.5)	6.25 (12.5)	>200 (>200)	–
*S. typhimurium* CCARM 8013^b^	>200 (>200)	50 (200)	6.25 (12.5)	25 (12.5)	3.12 (6.25)	>200 (>200)	–
*S. aureus* CCARM 3089^c^	>200 (>200)	50 (200)	12.5 (12.5)	50 (12.5)	1.56 (1.56)	–	>200 (>200)
*S. aureus* CCARM 3090^c^	>200 (>200)	50 (200)	12.5 (12.5)	25 (12.5)	3.12 (3.12)	–	>200 (>200)
*S. aureus* CCARM 3108^c^	>200 (>200)	50 (200)	12.5 (12.5)	50 (12.5)	1.56 (1.56)	–	>200 (>200)
*S. aureus* CCARM 3114^c^	>200 (>200)	50 (200)	12.5 (12.5)	25 (12.5)	3.12 (3.12)	–	>200 (>200)
*S. aureus* CCARM 3126^c^	>200 (>200)	50 (200)	12.5 (12.5)	25 (12.5)	1.56 (1.56)	–	>200 (>200)
Hemolysis^d^ (%)	0	0	8	71	100	–	–
GM^e^ (μM)	387.5	159.7	10.6	10.9	3.6	–	–
MHC^f^ (μM)	550	350	210	50	0.5	–	–
Therapeutic index^g^	1.4	2.1	**19.8** ^h^	4.5	0.1	–	–
Retention time^i^	17.6	19.5	21.7	23.8	–	–	–
Relative hydrophobicity^j^ (cationicity)^k^	28 (+3)^g^	31 (+4)^g^	34 (+5)^g^	38 (+6)^g^	–	–	–
Calculated^l^	645.7	960.1	1274.6	1588.9	–	–	–
Observed^l^	646.6	960.5	1274.9	1589.6	–	–	–

Antibacterial assays were performed in 10 mM sodium phosphate buffer, pH 7.2 and PBS, pH 7.2 (number in parentheses)

^a^Drug-resistant *Escherichia coli* strains

^b^Drug-resistant *Salmonella typhimurium* strains

^c^Drug-resistant *Staphylococcus aureus* strains

^d^Percent hemolysis with 200 μM peptide in PBS

^e^GM denotes the geometric mean of MIC values (in PBS) from all 16 microbial strains in this table. When no detectable antimicrobial activity was observed at 200 μM, a value of 400 μM was used for calculation of the therapeutic index

^f^The minimal peptide concentration [minimal hemolytic concentration (MHC)] that produces 10 % hemolysis

^g^Therapeutic index = MHC/GM. Larger values indicate greater antimicrobial specificity

^h^The boldface entries show the best peptide with broad-spectrum activity in terms of the therapeutic index against both Gram-negative and Gram-positive bacteria

^i^Retention time was measured using a C_18_ reverse phase analytical column (4.6 × 250 mm, 300 Å, 5 nm). The peptides were eluted over 60 min using a linear gradient of 5–60 % acetonitrile in water containing 0.05 % (v/v) trifluoroacetic acid

^j^Relative hydrophobicity is reflected by the percent of acetonitrile at the retention time (Rosenfeld et al. 2010)

^k^Cationicity (number in parentheses)

^l^Molecular weights of (KW)_*n*_ peptides

The antibacterial activities of the peptides in PBS and low ionic strength buffer [sodium phosphate (SP) buffer] are summarized in Table 1. In general, the antibacterial activity of the peptides increased with increasing chain length up to (KW)_4_. Whereas (KW)_2_ was inactive against all strains, (KW)_3_ and (KW)_4_ exhibited activity against *S. typhimurium*, *P. aeruginosa* and *L. monocytogenes*. Moreover, (KW)_4_ exhibited stronger antibacterial activity than the other peptides against all bacterial strains. Interestingly, the antibacterial activity of (KW)_5_ was similar to or somewhat weaker than that of (KW)_4_, indicating that, with (KW)_5_, increasing chain length did not lead to an increase in antibacterial activity.

**Fig. 1:**
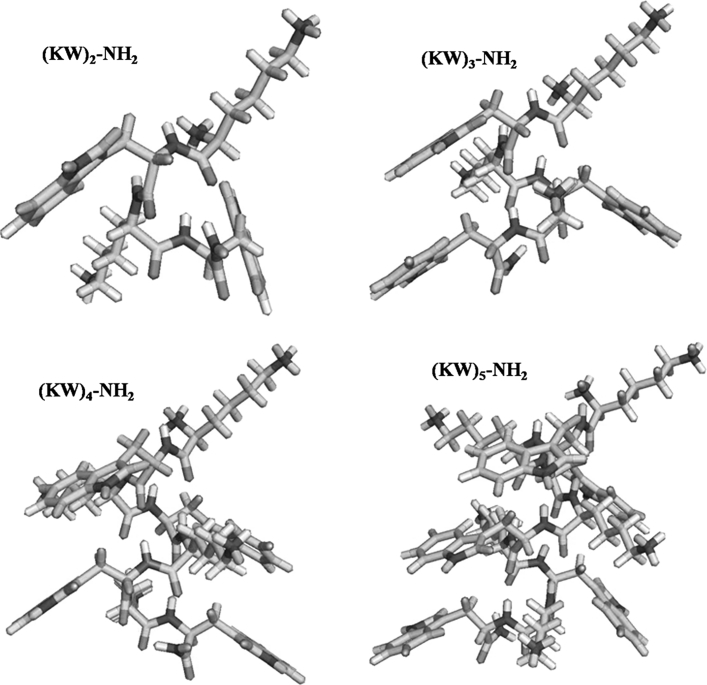
Model structures of (KW)_*n*_–NH_2_, the linear antibacterial peptides used in this study (*n* = 2, 3, 4 and 5). The peptide is shown as *cap-sticks*, with carbon atoms in *green*, nitrogen atoms in *blue*, and oxygen and hydrogen atoms in *red* and *white*, respectively (color figure online)

Neither (KW)_2_ nor (KW)_3_ induced hemolysis at a concentration of 200 μM (Table 1). In contrast, (KW)_4_ and (KW)_5_ caused 8 and 71 % hemolysis, respectively. The greater hemolytic activity of (KW)_5_ likely reflects its greater hydrophobicity. In addition, Feder et al. (2000) reported a linear correlation between hemolysis and the aggregation state. We therefore next examined the aggregation state of (KW)_5_ in aqueous solution. Computer modeling revealed that the clustering of KW motifs within (KW)_*n*_ peptides, resulted in different spatial arrangements and surface areas (Fig. 1).

### Structure and organization of (KW)_*n*_ peptides in aqueous solution

**Fig. 2:**
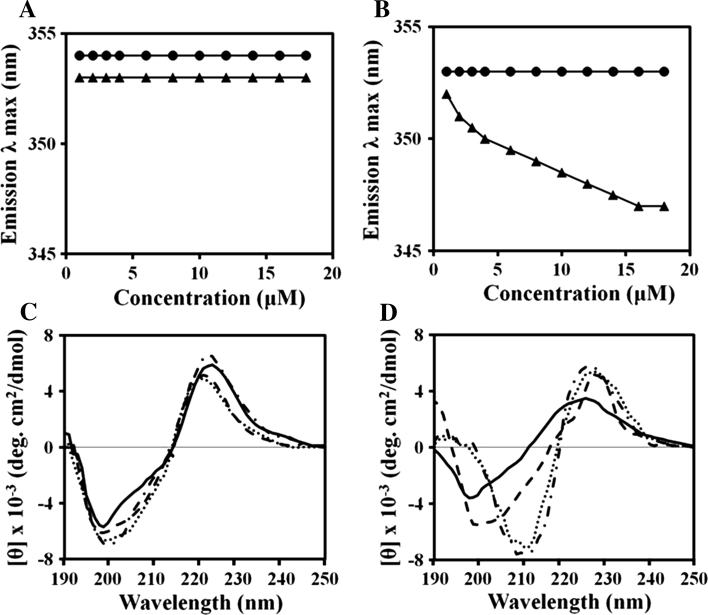
Structure and organization of (KW)_4_ and (KW)_5_ in aqueous solution. Aggregation states of the peptides in aqueous solution were determined based on Trp fluorescence and are shown as functions of the peptide concentration. The wavelength at the emission maximum was taken for plotting. (KW)_4_ (*filled circles*), (KW)_5_ (*filled triangles*): water (**a**) and PBS (pH 7.2) (**b**). Concentration-dependent CD spectroscopy was used to examine the conformations of the soluble and aggregation states of the peptides. Concentration-dependent CD spectra for (KW)_4_ (**c**) and (KW)_5_ (**d**) in PBS (pH 7.2): 25 μM (*solid line*), 50 μM (*dashed line*), 100 μM (*dotted line*) and 150 μM (*dashed-dotted line*)

To investigate why (KW)_5_ exhibited less antibacterial activity and greater hemolytic activity than (KW)_4_, we assessed the self-aggregation of (KW)_4_ and (KW)_5_ in water and PBS. As determined from the Trp fluorescence, neither peptide aggregated in water (Fig. 2a). In PBS, the Trp fluorescence maximum from (KW)_5_ dose dependently shifted from 352 to 340 nm due to its self-aggregation (Fig. 2b). On the other hand, the Trp fluorescence of (KW)_4_ did not change, implying no self-aggregation. We did not consider (KW)_2_ or (KW)_3_ in the self-aggregation assays because of their short chain lengths.

We further analyzed the aggregation states using CD spectroscopy. As shown in Fig. 2c and d, the peptides in PBS had a negative band at 200 nm, which is characteristic of random coil. These peptides also had a band at 225 nm due to the Trp side chain, which was consistent with earlier reports (Liu et al. 2007; Woody 1994; Ladokhin et al. 1997). Because (KW)_5_ assumes a random coil conformation, it failed to form a folded conformation at concentrations of 25 and 50 μM. At higher concentrations (100 and 150 μM), however, the negative band of (KW)_5_ was shifted to a slightly longer wavelength, suggesting that a small fraction of the peptide adopted a folded conformation or was weakly aggregated (Fig. 2d). In contrast, (KW)_4_ peptide maintained its random coil conformation in PBS at all tested concentrations (Fig. 2c).

### Localization of fluorescence-labeled peptides

**Fig. 3:**
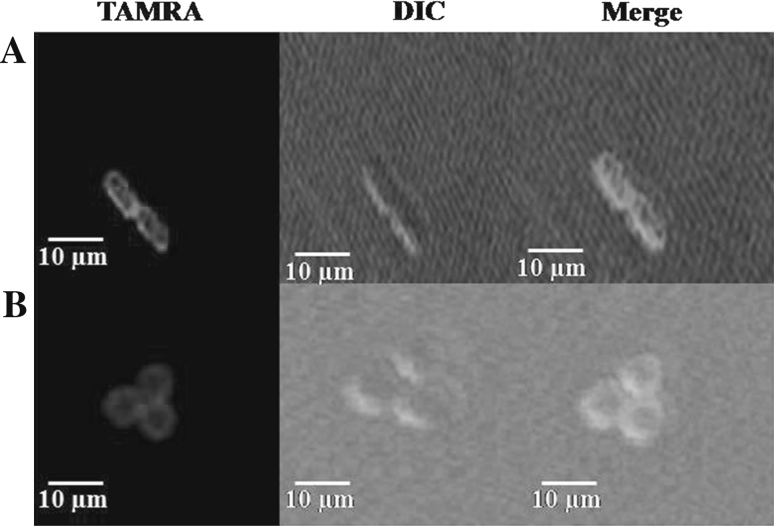
Confocal laser-scanning micrographs images of *E. coli* CCARM 1229 (**a**) and *S. aureus* CCARM 3090 (**b**) cells treated with TAMRA-labeled-(KW)_4_. The cells were treated with 12.5 μM TAMRA-(KW)_4_ for 10 min at 37° C in PBS (pH 7.2). From *left* to *right*: TAMRA, differential interference contrast (DIC), merged images

To determine the site targeted by (KW)_4_ in *E. coli* CCARM 1229 and *S. aureus* CCARM 3090, the bacteria were treated with the peptide and observed under CLSM. As shown in Fig. 3, the peptide bound to the surface of *E. coli* and *S. aureus* cells (Fig. 3), confirming that these peptides interact with the bacterial cell membrane.

### Interaction of peptides with LPS

**Fig. 4:**
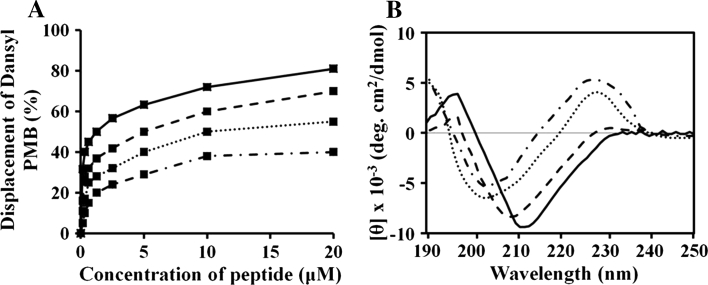
Binding affinities of the peptides for LPS, as measured using dansyl PMB displacement assays and CD spectroscopy. *P. aeruginosa* LPS (9 μg) was incubated with dansyl PMB (4 μM) for 5 min, after which fluorescence was measured at an excitation wavelength of 340 nm and emission wavelength of 485 nm. Peptides were added at different concentrations, and dansyl PMB fluorescence was measured after 5 min (**a**). CD spectra for the peptides (50 μM) were measured in the presence of 0.1 % LPS (**b**): (KW)_2_ (*dashed-dotted line*), (KW)_3_ (*dotted line*), (KW)_4_ (*dashed line*), and (KW)_5_ (*solid line*)

We next used dansyl PMB displacement assays to determine the binding affinity of each peptide for LPS. The method is based on enhanced dansyl fluorescence upon the interaction of LPS. This fluorescence is decreased when peptides bind to LPS and displace the dansyl PMB. As shown in Fig. 4a, all four tested peptides dose-dependently bound to LPS, with (KW)_5_ exhibiting the highest affinity. At a concentration of 20 μM, (KW)_5_ displaced 81 % of dansyl PMB from LPS. By comparison, (KW)_4_, (KW)_3_ and (KW)_2_ displaced 70, 55 and 40 %, respectively, at the same concentration. These data clearly indicate a correlation between chain length and LPS binding affinity.

We also used the peptides’ CD spectra to predict their interactions with LPS. When the peptides were studied at 50 μM in a 0.1 % LPS suspension, (KW)_2_ and (KW)_3_ exhibited no secondary structure, while the longer peptides adopted folded conformations (Fig. 4b), which is indicative of their strong binding to LPS and was highly correlated with their displacement of dansyl PMB.

### Membrane lytic and bactericidal activities of the peptides

**Fig. 5:**
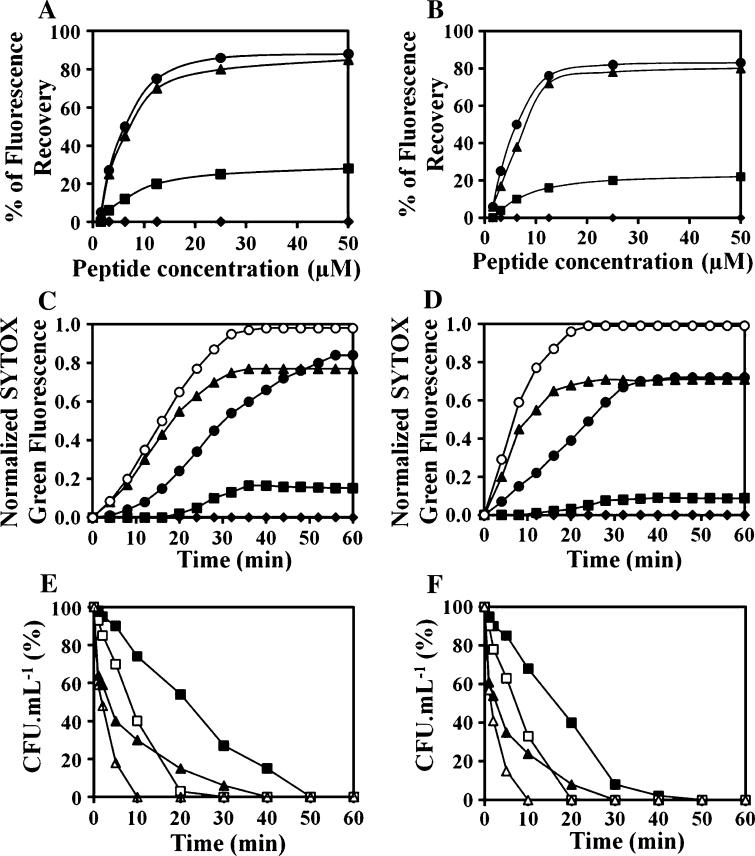
Membrane-disruption and bactericidal activity. Dose–response curves for peptide-induced membrane depolarization in intact *E. coli* CCARM 1229 (**a**) and *S. aureus* CCARM 3090 (**b**). Membrane depolarization was monitored as an increase in the fluorescence of DiSC_3_-5 (excitation wavelength 620 nm, emission wavelength 670 nm) after addition of various concentrations of peptide: (KW)_2_ (*filled diamonds*), (KW)_3_ (*filled squares*), (KW)_4_ (*filled circles*), and (KW)_5_ (*filled triangles*). The increase in fluorescence obtained using 0.1 % Triton X-100 was taken as 100 %. Membrane permeabilization was monitored based on entry of SYTOX Green dye. Bacterial cells (2 × 10^7^ CFU/ml) in PBS were incubated with 1 μM SYTOX Green dye. Peptides were added at a concentration of 12.5 μM, after which uptake of SYTOX Green through the *E. coli* (**c**) or *S. aureus* (**d**) plasma membrane was measured based on the time course of fluorescence changes at an excitation wavelength of 485 nm and emission wavelength of 520 nm: (KW)_2_ (*filled diamonds*), (KW)_3_ (*filled squares*), (KW)_4_ (*filled circles*), (KW)_5_ (*filled triangles*), and Melittin (*open circles*). Kinetics of the bactericidal activity against *E. coli* CCARM 1229 (**e**) and *S. aureus* CCARM 3090 (**f**). Bacteria treated with the respective peptides were diluted at the appropriate times and then plated on LB agar. CFUs were then counted after 16 h of incubation at 37° C. *Black* (KW)_4_, *white* (KW)_5_, *squares and triangles* 1 and 2 times the MIC, respectively; cells (2 × 10^5^ CFU/ml) incubated in the absence of any peptide served as controls

We examined the peptides’ capacity to disrupt the cytoplasmic membrane in *E. coli* and *S. aureus*. With the exception of (KW)_2_, the peptides dissipated the membrane potential of intact *E. coli* CCARM 1229 (Fig. 5a) and *S. aureus* CCARM 3090 (Fig. 5b) in a dose-dependent manner, and the longer peptides were more effective than the shorter ones. Moreover, the capacity of (KW)_4_ and (KW)_5_ to disrupt the cytoplasmic membrane correlated well with their antibacterial activities against the same bacteria (Table 1). Inactive (KW)_2_ did not depolarize the membrane, while (KW)_3_ elicited less depolarization than the longer peptides.

The ability of the (KW)_*n*_ peptides to permeabilize the cell membrane was determined based on the uptake of SYTOX Green, a fluorescent dye, by *E. coli* and *S. aureus* cells. After the addition of 12.5 μM peptide in PBS, influx of SYTOX Green fluorescence was measured for 60 min. Treatment with (KW)_4_ or (KW)_5_ efficiently activated uptake of the dye within 35 min in both *E. coli* (Fig. 5c) and *S. aureus* (Fig. 5d). (KW)_5_ induced internalization of SYTOX Green more quickly than (KW)_4_, which is indicative of its more efficient interaction and partition within the bacterial lipid bilayer, and is consistent with its greater hydrophobicity. (KW)_3_ elicited less uptake of SYTOX Green than (KW)_4_ or (KW)_5_. These results suggest that active peptides exert their antibacterial activities through induction of membrane damage.

To further investigate the bactericidal activities of (KW)_4_ and (KW)_5_, we performed a time-killing assay against *E. coli* CCARM 1229 and *S. aureus* CCARM 3090. As shown in Fig. 5e and f, at concentrations equal to or above the MIC, bacterial counts significantly declined within 35 min. (KW)_5_ killed bacteria more quickly than (KW)_4_, possibly due to its longer chain length and/or greater hydrophobicity, which is consistent with its faster permeabilization of the target bacterial membrane (Fig. 5c, d).

### Calcein release and turbidity assay in liposomes

**Fig. 6:**
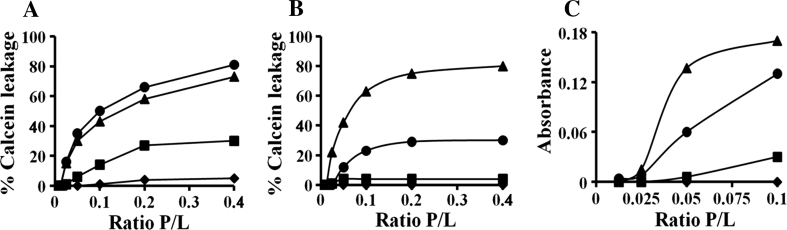
Peptide actions on lipid bilayers. Percent leakage of calcein from PE:PG (7:3, w/w) (**a**) and PC:CH (10:1, w/w) vesicles (**b**) at pH 7.4 was measured for 25 min after the addition of various doses of peptide. **c** LUV aggregation. Solutions containing various concentrations of peptide were added to a suspension of 400 μM PE:PG (7:3, w/w) LUVs, after which aggregation was monitored based on changes in the absorbance of the LUVs at 405 nm: (KW)_2_ (*filled diamonds*), (KW)_3_ (*filled squares*), (KW)_4_ (*filled circles*), and (KW)_5_ (*filled triangles*)

We also evaluated the abilities of these peptides to cause leakage of entrapped calcein from PE:PG (7:3, w/w) LUVs, which mimic a bacterial membrane. At a lipid-to-peptide molar (L/P) ratio of 2.5:1, (KW)_4_ released 81 % of entrapped calcein, while (KW)_5_ released about 73 %. (KW)_3_ exhibited moderate activity, but (KW)_2_ was inactive (Fig. 6a). We also examined the leakage of entrapped calcein from using zwitterionic liposomes composed of PC:CH (10:1, w/w), which mimic eukaryotic membranes. At a L/P ratio of 2.5:1, (KW)_5_ and (KW)_4_ released 80 and 30 % of entrapped calcein, respectively (Fig. 6b), while (KW)_2_ and (KW)_3_ elicited no release of calcein from zwitterionic vesicles. The peptides’ respective abilities to release dye from PC:CH vesicles is therefore consistent with their hemolytic activities (Table 1).

We then measured changes of liposome turbidity to monitor the effects of the peptides on the size of PE:PG (7:3, w/w) vesicles. We found that the peptides increased the turbidity of PE:PG (7:3, w/w) vesicles, indicating an increase in vesicle size, most likely due to vesicle lysis and aggregation (Fig. 6c) (Domingues et al. 2009).

### Characterization of the Trp environment using fluorescence spectroscopy

To characterize the Trp environment of the peptides, we used fluorescence spectroscopy to examine the binding of the peptides to lipid bilayers. Trp emission spectra was recorded in the presence of negatively charged PE:PG (7:3, w/w) or zwitterionic PC:CH (10:1, w/w) vesicles or in PBS (Table 2). For all of the peptides except (KW)_5_, the fluorescence maximum in buffer was around 353 nm, indicating that the Trp residues were situated in a more hydrophilic environment. For (KW)_5_, the fluorescence spectra suggest that Trp is shifted from a polar to less polar environment, possibly due to self-aggregation of the peptide in PBS (Fig. 2).

**Table 2:** Tryptophan emission maxima of 2 μM peptides and *K*_SV_ in PBS (pH 7.2) or in the presence of 200 μM PE:PG (7:3, w/w) SUVs and 200 μM PC:CH (10:1, w/w) SUVs

Peptides	λ_max_ buffer (nm)	Blue shift (nm)		*K*_SV_ (M^−1^)^a^		
		PE:PG (7:3, w/w)	PC:CH (10:1, w/w)	Buffer	PE:PG (7:3, w/w)	PC:CH (10:1, w/w)
(KW)_2_	353	8	2	15	2.8	5.8
(KW)_3_	353	11	2	15	2.3	5.3
(KW)_4_	353	17	5	14	1.6	5.1
(KW)_5_	351	14	9	11	2.0	4.3

^a^ *K*_SV_ is the Stern–Volmer constant. *K*_SV_ (M^−1^) were determined from the Stern–Volmer equation *F*_0_/*F*_1_ = 1 + *K*_SV_ (*Q*), where *Q* is the concentration of quencher (acrylamide). Concentration of the quencher varied from 0.04 to 0.20 M

The ability of the peptides to bind to PE:PG (7:3, w/w) vesicles was in the order: (KW)_4_ > (KW)_5_ > (KW)_3_ > (KW)_2_. In addition, (KW)_4_ exhibited a large blue shift, indicating that its Trp side chains partitioned preferentially into a more rigid, and hydrophobic environment within the PE:PG (7:3, w/w) vesicles. Similarly, the Trp emission from (KW)_5_ (342 nm) also exhibited a blue shift in the presence of PC:CH (10:1, w/w) vesicles, indicating migration of Trp residues to a more hydrophobic environment (Burstein et al. 1973). On the other hand, no blue shifts were detected for (KW)_2_, (KW)_3_ or (KW)_4_ in the presence of the more hydrophobic environment of PC:CH (10:1, w/w) vesicles.

We evaluated the ability of Trp residues to access the lipid bilayer by measuring the Stern–Volmer quenching constants (*K*_SV_) using soluble acrylamide as a quencher. Free Trp residues in aqueous solution were fully quenched by acrylamide (*K*_SV_ = ~9 M^−1^) (Wimley et al. 1996), and the *K*_SV_ values for (KW)_2_, (KW)_3_, (KW)_4_ and (KW)_5_ in PBS were (approximately) 15, 15, 14, and 11 M^−1^, respectively (Table 2). That the *K*_SV_ value for (KW)_4_ was 1.6 M^−1^ in the presence of PE:PG (7:3, w/w) vesicles, indicates that the Trp residues of (KW)_4_ were more protected in PE:PG (7:3, w/w) vesicles than PC:CH (10:1, w/w) vesicles (Table 2). This tendency correlated well with the potent antibacterial activity of (KW)_4_. In contrast, the hemolytic peptide (KW)_5_ had a lower *K*_SV_ value, indicating that its Trp residues were more anchored within the hydrophobic core of the zwitterionic phospholipids, which correlates well with the strong induction of calcein leakage from the vesicles (Fig. 6b).

### Structures of the peptides in liposomes

**Fig. 7:**
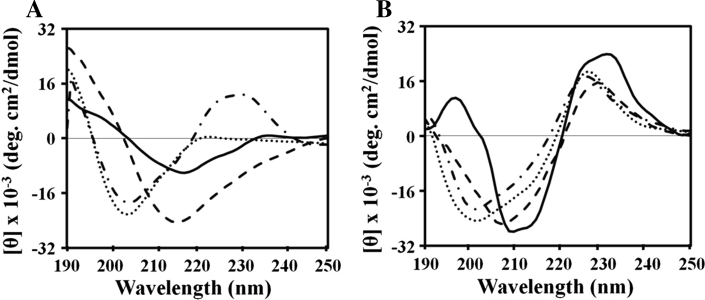
CD spectra for (KW)_2_ (*dashed-dotted line*), (KW)_3_ (*dotted line*), (KW)_4_ (*dashed line*), and (KW)_5_ (*solid line*) in the presence of PE:PG (7:3, w/w) (**a**) and PC:CH (10:1, w/w) vesicles (**b**)

We determined the secondary structures of the peptides in aqueous solution and lipid membranes. (KW)_2_ and (KW)_3_ displayed no secondary structure in aqueous solution (data not shown) or lipid membranes. (KW)_4_ and (KW)_5_ adopted folded conformations on liposomes (Fig. 7). (KW)_5_ displayed a positive band at 197 nm and a negative band at 210 nm on PC:CH vesicles (Fig. 7b), indicating that its side chain partitioned preferentially into the more rigid and hydrophobic environment of the lipid bilayer, which is consistent with its potent hemolytic activity.

### Observation of (KW)_4_ activity in *E. coli* using SEM

To examine the action of (KW)_4_ against *E. coli* CCARM 1229 in more details, we used SEM to observe the morphological changes that occur upon incubation of the peptide with bacteria. In the absence of peptide, the bacteria had a smooth surface (Supplementary Fig. 1A), but in the presence of 12.5 μM peptide the treated bacteria appeared clumped with more crenated surfaces (Supplementary Fig. 1B).

### Bacterial agglutination

To confirm the bacterial aggregation, we incubated *E. coli*, *S. aureus*, or *P. aeruginosa* with various concentrations of peptide for 1 h. At higher concentrations of peptide, there was greater agglutination and bacterial clumping (Supplementary Fig. 2). (KW)_4_ induced agglutination of all three bacterial strains. Bacterial aggregation was not observed in the absence of peptide. These results demonstrate the agglutination-inducing activity of (KW)_4_ peptide against both Gram-negative and Gram-positive bacteria.

## Discussion

De novo design of short peptides is an alternative strategy for the development of AMPs that are selective for bacteria, with little or no effect on human cells. We designed peptides comprised of alternating Lys and Trp residues, which we previously showed to have antimicrobial activity (Gopal et al. 2011). When choosing a cationic amino acid for the present study, we chose Lys over Arg because, the guanidinium group in the side chain of Arg is active in both microbial and mammalian cell membranes (Yang et al. 2003). Lys also possesses one positive charge but lacks a guanidinium group, making it less toxic to mammalian cells (Andra et al. 2007). In addition, the cost of producing Arg-containing peptides is higher than that of producing Lys-containing ones, which is an important factor when considering drug design and development. Finally, Trp was selected, based on reports indicating that AMPs containing Trp are more active than those containing either Tyr or Phe (Dathe et al. 2004; Strom et al. 2003; Subbalakshmi et al. 2000). One explanation could be that the bulkier side chain of Trp may interact more strongly with the hydrophobic membrane core than other non-polar amino acids, such as Phe and Tyr (Strom et al. 2002; Wimley and White 1996).

The results obtained in earlier studies of the effect of chain length on the activity of AMPs have varied. In one study of a (RW)_*n*_ series, chain length correlated well with antibacterial and hemolytic activities (Liu et al. 2007). But in another study of H-(LARL)_3_-(LRAL)_*n*_ peptides, increasing chain length reduced the antibacterial activity while increasing hemolysis (Niidome et al. 2005). In the present study, we observed that antibacterial activity improved with increasing chain length up to (KW)_4_; there was no further increase in antibacterial activity when the chain length exceeded eight amino acids. The order of the activity was (KW)_2_ < (KW)_3_ < (KW)_4_ > (KW)_5_. (KW)_5_ exhibited nearly the same antibacterial activity as (KW)_4_, except when treating *S. typhimurium* and *P. aeruginosa* in PBS. The observed increase in antibacterial activity as the peptide chain lengthened is attributable to increases in the cationicity, hydrophobicity and clustering of the KW motifs (Fig. 1). When chain length exceeded the optimum eight residues, as with (KW)_5_, antibacterial activity declined, and there was a greater propensity for aggregation in aqueous buffer due to the increased hydrophobicity. The observed difference in the peptides’ ability to aggregate in PBS and SP buffer may be attributable to differences in the ionic strengths of the two buffers. Our results suggest that (KW)_5_ was soluble and weakly self-aggregated in PBS (Fig. 2).

The hemolytic activities of the (KW)_*n*_ peptides increased with chain length. Short peptides were inactive, and (KW)_4_ showed modest hemolytic activity at high concentrations. (KW)_5_ exhibited marked hemolytic activity, reflecting an increase in hydrophobicity as well as the peptide’s ability to weakly aggregate. Several studies have suggested that AMPs in a self-aggregated state in aqueous solution, where there is an increase in hydrophobic interactions, mediate hemolytic activity (Feder et al. 2000; Javadpour and Barkley 1997). Our results with (KW)_5_ are consistent with that idea. Only (KW)_4_ exhibited good antibacterial activity without a high degree of hemolysis. This may reflect an optimal balance of cationicity and hydrophobicity, which are features needed for broad-spectrum antibacterial activity without hemolysis (Javadpour et al. 1996; Fernandez-Lopez et al. 2001).

AMPs kill bacteria either through permeabilization of the cytoplasmic membrane or by binding with intracellular targets like nucleic acids (Brogden 2005). Our CLSM results indicate that (KW)_*n*_ peptides interact with the bacterial surface (Fig. 3), suggesting (KW)_*n*_ act by damaging the cytoplasmic membrane, rather than binding to nucleic acids. Gram-negative bacteria have two-cell membranes, the outer membrane and inner cytoplasmic membrane. The outer leaflet of the outer membrane consists of LPS, making it necessary for the peptide to bind to LPS and cross the outer membrane before entering the periplasmic space (Farnaud et al. 2004; Bhattachariya and Ramamoorthy 2009). For this reason, we examined the peptides’ ability to bind LPS. We found that longer peptides strongly bound LPS (Fig. 4) and induced membrane depolarization (Fig. 5a, b). The higher affinities of (KW)_4_ and (KW)_5_ for LPS correlated with their abilities to induce membrane depolarization, suggesting they effectively disrupted the outer membrane and accessed the cytoplasmic membrane. The peptides then appeared to damage the inner membrane (Fig. 5c, d) to exert their bactericidal action (Fig. 5e, f).

Using lipid bilayers, it was previously shown that the mechanism of action of AMPs involves two stages: initial electrostatic interaction of the cationic residues (Lys or Arg) of the peptides with the anionic lipids of the bacterial membrane (Juffer et al. 2001; Shepherd et al. 2001), followed by hydrophobic interaction of aromatic side chain-containing amino acids with the lipid bilayer (Schibli et al. 2002; Wimley and White 1996). Our results are consistent with that mechanism. The observed Trp blue shifts and *K*_SV_ values (Table 2) indicate that for all of the (KW)_*n*_ peptides tested, the Trp residues partitioned into the lipid bilayer of PE:PG (7:3, w/w) vesicles. The smallest peptide of the series, (KW)_2_, induced no calcein leakage or aggregation (Fig. 6), which likely reflects this peptide’s inability to permeate the bacterial membrane. (KW)_3_ exhibited a greater ability to bind LPS and induce membrane permeabilization, calcein leakage and aggregation than (KW)_2_, but its effects were still considerably weaker than those of (KW)_4_ and (KW)_5_. Furthermore, (KW)_2_ and (KW)_3_ lacked definite secondary structure on lipid membranes, as evidenced by their CD spectra. Briefly, the CD spectra showed that at a P/L ratio of 1:50, (KW)_4_ and (KW)_5_ formed stable structures within the negatively charged lipid membrane, suggesting interfacial association of the peptides (Fig. 7). Clearly, peptide length, hydrophobicity and charged/hydrophobic surface ratio correlate with the degree of membrane disruption/permeabilization. The exception is (KW)_5_, which showed less antibacterial activity than (KW)_4_, though its chain length is longer. This may reflect the weak aggregation of (KW)_5_ in aqueous solution, which would reduce its electrostatic potential and therefore its antibacterial activity. Our Trp blue shift assays and quenching experiments showed that its aggregation state led (KW)_5_ to bind more weakly to membranes than did (KW)_4_, which would also reduce its ability to permeabilize the membrane. Consistent with this interpretation, the degree of membrane disruption is reportedly dependent on the concentration of peptide adsorbed onto the negatively charged lipid membrane (Ringstad et al. 2007).

Many Trp-rich AMPs are not cell type-selective (Subbalakshmi et al. 1996; Ahmad et al. 1995; Schibli et al. 2002). In the present study, only (KW)_5_ caused significant leakage from PC:CH liposomes, and only (KW)_5_ exhibited a large blue shift, which indicates that only (KW)_5_ entered the hydrophobic core of the mammalian membrane. Our results also show that (KW)_*n*_ peptides bind more tightly to bacterial membrane-mimicking liposomes than to those mimicking a mammalian membrane. This is because bacterial membranes consist of almost 25 % anionic lipids, whereas mammalian membranes contain large amounts of PC lipids, with negligible amounts of anionic lipids (Verkleij et al. 1973). Anionic lipids promote the initial electrostatic interaction between positively charged AMPs and the negatively charged membrane. Because the mammalian membrane is deficient in anionic lipids, AMPs cannot undergo a proper initial interaction. Infact, the positive charge of PC lipids may actually repulse the basic residues of AMPs. These results are consistent with an earlier study (Liu et al. 2007), which confirmed that (RW)_*n*_ peptides strongly interact with PG-containing bilayers, but possess only moderate affinity toward mammalian model membranes. However, it is the hydrophobic interaction between AMPs and the lipid bilayer core that is essential for membrane disruption. Consequently, it is the more hydrophobic AMPs that lack cell type selectivity and cause more damage to membranes (Tossi et al. 2000; Makovitzki et al. 2006). Similarly, highly aggregated peptides having high hydrophobicity reportedly induce fatal damage to mammalian membranes (Biggs et al. 2007). The hemolysis and evoked calcein release seen with (KW)_5_ are consistent with that finding.

In addition to causing membrane damage, (KW)_4_ also elicited bacterial agglutination. Liposome agglutination is dependent on the number of electrostatic/hydrophobic interactions with the peptides, which in turn depends on the number of Lys/Trp residues (Stromstedt et al. 2009). Our data show that several Lys and Trp residues are required for permeabilization of the bacterial membrane and agglutination of model liposomes. Acrylamide quenching studies corroborate those results, as longer peptides permeated deeper into the lipid bilayer core than shorter ones (Table 2). The formation of macroscopic aggregates involves at least two steps: peptide-membrane association with concomitant charge neutralization followed by vesicle association (Cummings and Vanderlick 2007; Fujii et al. 1993). In addition to altering surface charge density upon adsorption to the membrane, peptides dehydrate lipid head groups and induce aggregation of phospholipid vesicles. Reducing charge density also reduces peptide–peptide long-distance repulsion, which facilitates the formation of hydrophobic interactions between peptides associated with adjacent bilayers (De Meulenaer et al. 1997). Other studies found that more highly charged residues, and neutralization of those charges may be required to induce fusion between giant liposomes (Nomura et al. 2004). It has also been reported that membrane destabilization is essential for fusion (Ryan et al. 2005 and Domingues et al. 2009), and that peptides and proteins forming hydrophobic or hydrophilic cationic clusters show membrane-fusion activity (Cummings and Vanderlick 2007; Niidome et al. 2000; Fujii et al. 1993; Abu-Ghazaleh et al. 1992). In the present study, (KW)_4_ and (KW)_5_ damaged membranes and caused vesicles aggregation/fusion (Fig. 6). Because (KW)_*n*_ peptides contain only Lys and Trp residues, they can engage in both electrostatic and hydrophobic interactions with anionic lipid membranes and induce membrane destabilization and agglutination when dispersed on negatively charged vesicles.

The ability to cause vesicle leakage and aggregation was first identified with seminal ribonuclease (Mancheno et al. 1994) and various other synthetic peptides (Stromstedt et al. 2009), including K7, R7, K7W7 and R7W7. In the present study, (KW)_4_ destroyed the *E. coli* membrane (Supplementary Fig. 1) and caused agglutination of the bacterial cells (Supplementary Fig. 2). In addition to cell–cell aggregation, an extracellular matrix-like material was observed within the cell agglomeration. Proteins or peptides do not induce spontaneous aggregation of bacterial cells (Jung et al. 2009). Instead, agglutination results from peptide–charge and peptide–lipid interactions, which most likely involves hydrophilic/hydrophobic interactions with bacterial membranes (Gorr et al. 2008). Our finding that LPS-deficient Gram-positive bacteria, as well as liposomes, showed agglutination upon peptide treatment suggests that LPS does not play a role in bacterial agglutination.

Other studies have shown that RNase 3 triggers vesicle aggregation followed by membrane disruption, whereas RNase 7 induces dye leakage before aggregation (Torrent et al. 2009). Our SYTOX Green assays showed that (KW)_4_ and (KW)_5_ caused membrane destabilization at their MIC. In addition, the membrane depolarization assays showed that the peptides induced outer membrane permeabilization at concentrations lower than the MIC, and the time-frame of the effects of (KW)_4_ and (KW)_5_ on membrane permeability was consistent with the peptides’ bactericidal activities. However, (KW)_4_ induced bacterial agglutination at significantly higher concentrations than were needed to cause membrane permeabilization and bactericidal activity. This suggests that cell aggregation occurs after bacterial killing, and the initial step in the mechanism involves membrane disruption. Consistent with that idea, it is widely believed that membrane disruption is the primary mechanism by which AMPs exert their bactericidal effects (Hancock 2001; Brogden 2005; Tossi et al. 2000; Shai 2002). Induction of agglutination would help to clump bacterial cells together, which would increase their susceptibility to phagocytosis (Gorr et al. 2008). The ability of (KW)_4_ to bind to the bacterial membrane and induce agglutination makes it a potential candidate for future optimization.

**Fig. 8:**
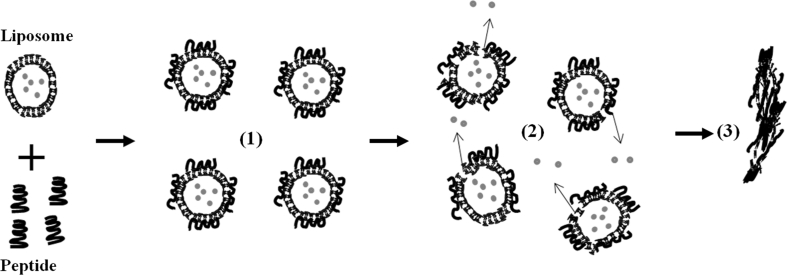
Schematic representation of the proposed mechanism of action of (KW)_*n*_ peptides in bacterial cells. (*1*) The peptide molecules associate with cell membranes or lipid bilayers, (*2*) the membrane/bilayer is disrupted, releasing the internal contents of the cell/vesicles; and (*3*) although higher peptide-lipid ratios necessary for cells/vesicles aggregate

In conclusion, the steps of the proposed mechanism by which (KW)_*n*_ peptides exert their bactericidal effects are as follows (Fig. 8): (1) The peptide binds to membrane phospholipid polar heads through electrostatic interactions. (2) The hydrophobic residues promote peptide–lipid interactions, the clustering of cell/liposomes–peptide complexes and destabilization of the lipid bilayer. (3) Cell/liposome aggregation. Membranolytic peptides with agglutinating properties may eventually represent as a new class of short antibacterial peptides useful for killing bacteria resistant to other types of antibiotics.

## Electronic supplementary material

Below is the link to the electronic supplementary material.

Supplementary material 1 (DOCX 282 kb)

## Abbreviations

CD: Circular dichroism
CH: Cholesterol
CFU: Colony forming units
DiSC_3_-5: 3,3′-Diethylthiodicarbocyanine iodide
PC: l-*α*-Phosphatidylcholine
PE: l-*α*-Phosphatidylethanolamine
PG: l-*α*-Phosphatidylglycerol
Fmoc: Fluoren-9-ylmethoxycarbonyl
hRBCs: Human red blood cells
MIC: Minimal inhibitory concentration
PMB: Polymyxin B
SUVs: Small unilamellar vesicles
TAMRA: 5-Carboxytetramethylrhodamine
