# Supplementary material for: Effect of repetitive lysine–tryptophan motifs on the bactericidal activity of antimicrobial peptides
Source: Amino Acids. 2012 Aug 23;44(2):645–60. doi: 10.1007/s00726-012-1388-6 (PMC3549253; doi:10.1007/s00726-012-1388-6)
Supplement: Supplementary file 1 — Supplementary material 1 (DOCX 282 kb) [file 726_2012_1388_MOESM1_ESM.docx]

**Electronic supplementary materials**

**Fig. S1** Scanning electron micrographs of *E. coli* CCARM 1229 cells in the absence (**A**) or presence (**B**) of (KW)_4_ peptide (12.5 μM). Bacteria were incubated with the peptide for 1 h at 37°C in PBS (pH 7.2).

**
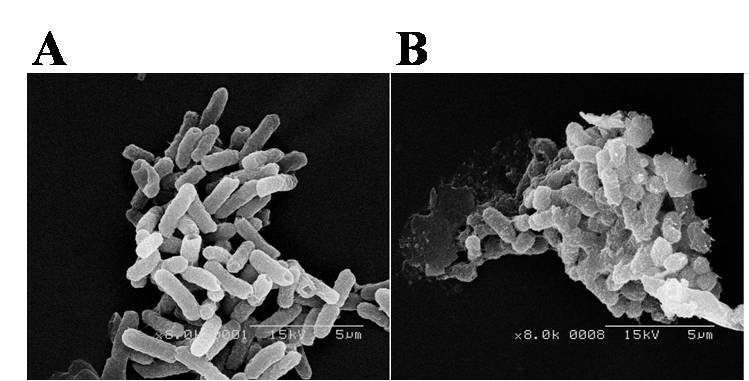
**

**Fig. S2** (KW)_4_-induced bacterial agglutination. Peptides were incubated with 2 x 10^8^ CFU/ml of *E. coli*, *S. aureus or P. aeruginosa* for 60 min at 37°C, after which bacterial aggregates were stained with 0.1% crystal violet and the supernatants removed. The aggregated bacteria remaining on the bottom of the plate were observed under an inverted microscope. Bar size is 200 μm.**
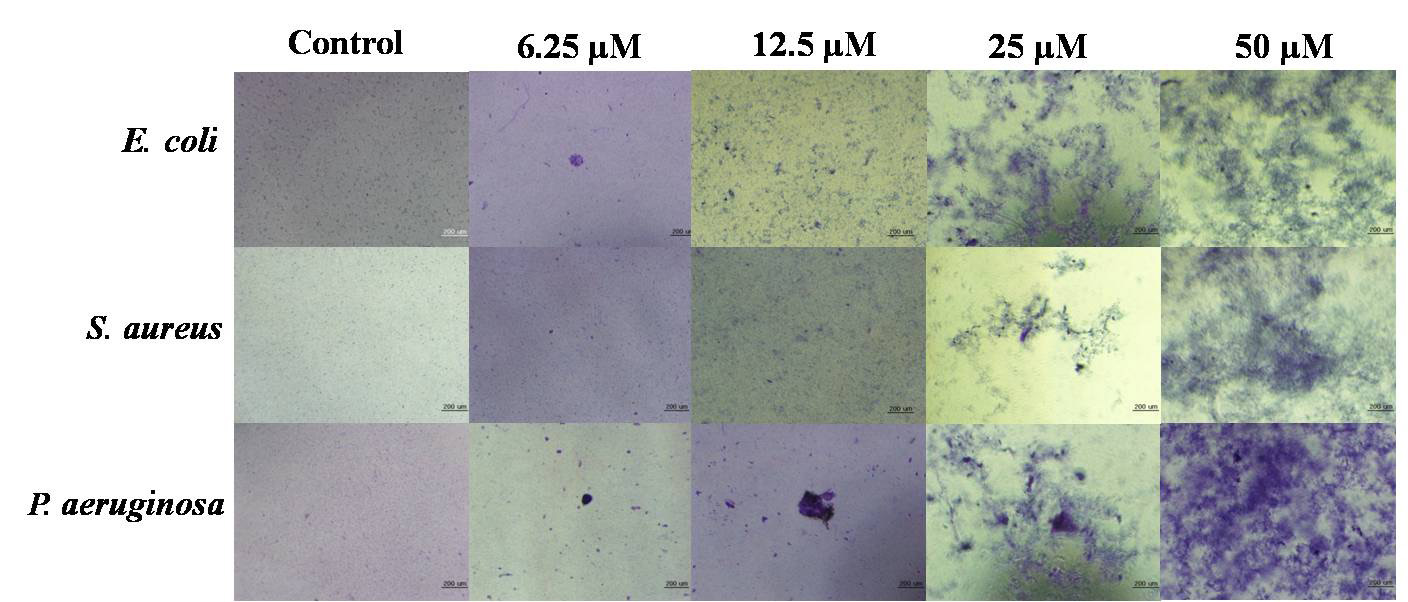
**
